# Intraparenchymal Enzyme Injections in Islet Isolations With Incomplete Ductal Perfusion of Enzymes

**DOI:** 10.3389/ti.2025.13507

**Published:** 2025-04-30

**Authors:** Maarten C. Tol, Dirk-Jan Cornelissen, Yun Suk Chae, Ezra J. van der Wel, Jeroen C. Sijtsma, Jason B. Doppenberg, Corine J. Vermeulen, Maaike A. J. Hanegraaf, Evelien H. van Rossenberg, J. Sven D. Mieog, Bert A. Bonsing, Volkert A. L. Huurman, Eelco J. P. de Koning, Marten A. Engelse

**Affiliations:** ^1^ Department of Internal Medicine, Leiden University Medical Center, Leiden, Netherlands; ^2^ LUMC Transplant Center, Leiden University Medical Center, Leiden, Netherlands; ^3^ Department of Surgery, Leiden University Medical Center, Leiden, Netherlands

**Keywords:** intraparenchymal injection, pancreatic islet, islet isolation, digestion, collagenase

Dear Editors,

Pancreatic islet isolation relies on the complete perfusion of digestive enzymes throughout the pancreas for dissociation of the extracellular matrix to digest pancreatic tissue and maximize the islet yield [1–3]. Enzymes are perfused in the pancreatic duct by retrograde cannulation (RC) or through combined ante- and retrograde cannulation (ARC) by dissecting the pancreas at the neck [4, 5]. Incomplete enzyme perfusion is often observed in pancreases of patients with chronic pancreatitis undergoing total pancreatectomy with islet autotransplantation (TPIAT) and at the dissection surface during ARC procedures. Here we describe intraparenchymal injections (IPI) of digestive enzymes as a potential solution to overcome incomplete perfusion.

All data were collected on consecutive human pancreatic islet isolations for clinical use between December 2014 and February 2024 in the Leiden University Medical Center. Pancreases for allogeneic islet transplantation were allocated by Eurotransplant. Pancreases for autologous islet transplantation were obtained after total pancreatectomy. Islet isolations were performed using an adapted version of the semi-automated method [4, 6]. RC was the standard method of cannulation, ARC was used if RC proved challenging. Experienced members of the islet isolation team examined the pancreas for hypoperfused tissue areas and performed intraparenchymal injections of digestive enzymes using 25–30 gauge needles until those areas were distended. IPI is demonstrated in Supplementary Video S1. Further details are provided in the Supplementary Methods.

Data from 253 consecutive islet isolations from donor pancreases intended for allogeneic islet transplantation, and 26 islet isolations from pancreases intended for autologous islet transplantation were included. Allogeneic organ donors had a mean age of 47.9 ± 12.7 years, 45.5% were female, and the body mass index was 27.3 ± 5.1 kg/m^2^. In procedures involving donor pancreases, RC was performed in 218 (86.2%) and ARC in 35 (13.8%) of the isolations (Supplementary Table S1). Patients with an indication for total pancreatectomy and islet autotransplantation had a mean age of 45.5 ± 14.9 years, 65.4% were female, the body mass index was 24.0 ± 4.1 kg/m^2^, and 84.6% had a history of chronic pancreatitis (Supplementary Table S2)

In islet isolations for autologous transplantation, digestion with IPI was higher (IPI 81.4% ± 15.5% vs without IPI 55.0% ± 27.4%, 95% CI of change: 7.82–45.02, p = 0.01, Figure 1D). Median islet yield was 5,540 (IQR 3,100–7,330) IEQ/g with IPI and 2,570 (IQR 1,870–3,230) IEQ/g without IPI (p = 0.05, Figure 1E) (Supplementary Table S3). We found that ductal cannulation with enzyme perfusion was not possible in 6 of these islet isolations. In these 6 isolations, we performed intraparenchymal enzyme injections only and isolated between 190.000 and 705.000 IEQ (range 2972–9503 IEQ/kg, Figures 1F, G; Supplementary Table S4). Five out of 6 islet preparations were transplanted. One of these islet products could not be transplanted because of a high endotoxin concentration.

**FIGURE 1:**
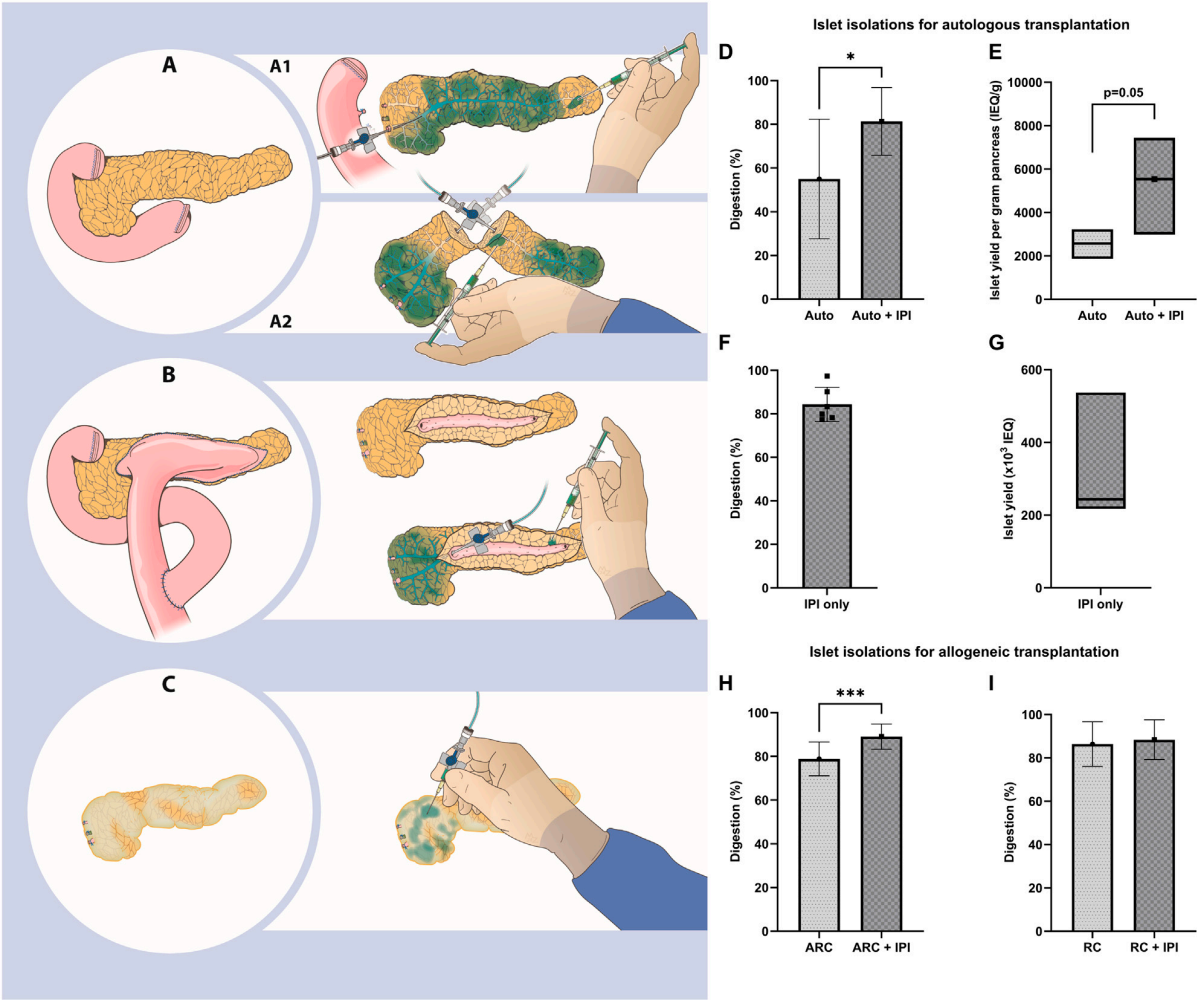
**(A)** The starting point for islet isolation with the pancreas connected to the duodenum. A1) The pancreas is separated from the duodenum and perfused by retrograde cannulation (RC) of the pancreatic duct. Certain tissue areas are poorly perfused and intraparenchymal injections (IPI) are performed to deliver enzymes (depicted in blue). A2) The pancreas is cut at the neck and enzymes are perfused by ante- and retrograde cannulation (ARC) of the pancreatic duct. Parenchyma around the cut surface is poorly perfused and IPI is performed. **(B)** A pancreas after pancreaticojejunostomy. The jejunum is removed and the pancreatic duct exposed. Enzymes are perfused by ARC and IPI. **(C) **A smaller, fibrotic pancreas. Ductal cannulation is not possible and IPI is performed. **(D-G)** Islet isolations for autologous transplantation. **(D)** Percentage digestion (mean ± standard deviation). **(E)** islet yield per gram pancreas (median and interquartile range).** (F)** Percentage digestion (mean ± standard deviation) and **(G)** islet yield (median and interquartile range) using only IPI. **(H, I)** Islet isolations for allogeneic transplantation showing percentage digestion (mean ± standard deviation) for ARC **(H)** and RC **(I)**. IEQ, islet equivalent.

In islet isolations for allogeneic transplantation, we found a higher digestion in ARC isolations with IPI of 10.0%pt. (95% CI: 5.99–14.08, p < 0.001, Figure 1H), without a difference in islet yield per gram pancreas. For RC islet isolations, digestion and islet yield per gram pancreas were similar between the isolations with and without IPI (Figure 1I).

Generation of a maximal number of viable and functional islets is the most important goal for islet isolation. In this observational study we show that intraparenchymal injection is unlikely to have a negative effect on islet yield. The potential contribution of intraparenchymal enzyme injections was demonstrated in 6 isolations for autologous islet transplantation with a sufficient islet yield for autotransplantation. Digestion rate and islet yield of isolations using ARC in donor pancreas and of isolations for TPIAT were higher when IPI was performed based on the presence of hypoperfused pancreas parenchyma. In RC isolations, similar digestion and islet yield were present.

IPI could be considered in pancreases with an altered anatomy, such as after dissection of the neck for ARC (Figure 1A) and after previous pancreatic surgery. Damage to the pancreas due to dissection, which is inherent to ARC, leads to hypoperfusion and subsequent incomplete digestion. Fibrosis, calcification (Supplementary Figures S1A, C) and previous surgery (e.g., Frey, Beger, Puestow procedures; Figures 1B, C) are often present when pancreases are presented for isolation in the context of autologous islet transplantation [7]. These surgical procedures may render classical perfusion methods inadequate and negatively affect islet yield [8]. In these instances, intraparenchymal injections may facilitate more complete perfusion of the parenchyma with digestive enzymes, potentially supporting digestion and islet yield.

There are no previous studies on how to deal with hypoperfused pancreatic parenchyma during isolation. A strength of this study is the inclusion of consecutive islet isolations of pancreases for both allogeneic and autologous islet transplantation. Study limitations include its retrospective, observational nature and judgement of hypoperfusion by experienced members of the islet isolation team. In order to obtain more robust information of the contribution of IPI on islet isolation outcome, randomized studies with or without IPI, and more objective assessment of hypoperfusion should be performed.

In conclusion, intraparenchymal injections may improve digestion and islet yield, representing a potential addition to current islet isolation practice.

## Data Availability

The datasets presented in this article are not readily available because instutition-guided restrictions may be applicable. Requests to access the datasets should be directed to ME, m.a.engelse@lumc.nl.
