# Supplementary figures and images for: Intraparenchymal Enzyme Injections in Islet Isolations With Incomplete Ductal Perfusion of Enzymes
Source: Transpl Int. 2025 Apr 30;38:13507. doi: 10.3389/ti.2025.13507 (PMC12074925; doi:10.3389/ti.2025.13507)

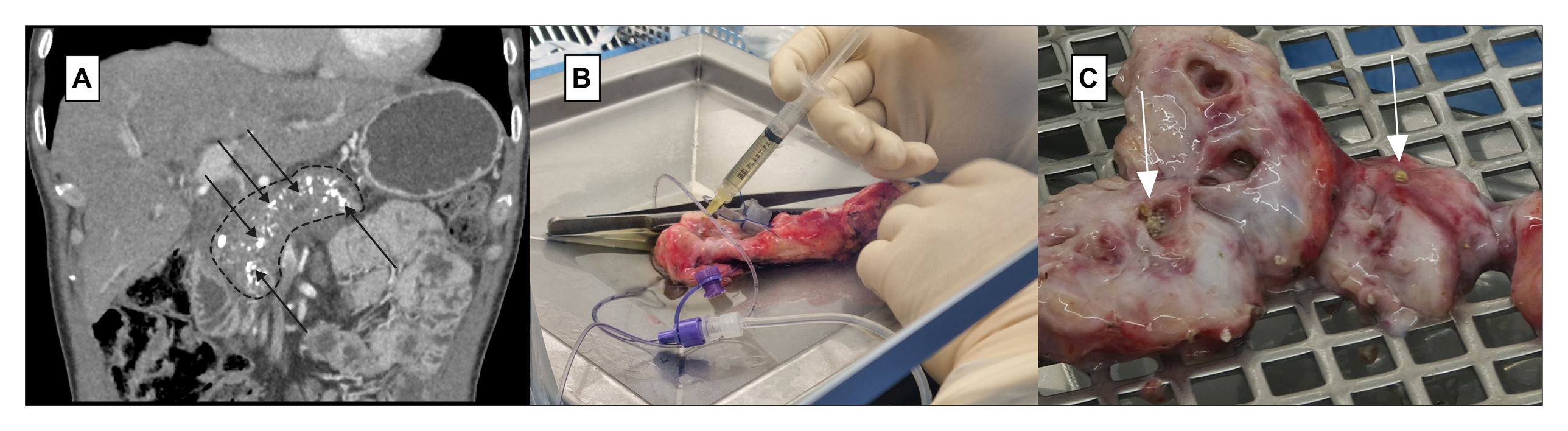

Supplement: Supplementary file 1 [file Image1.TIF]
