# Supplementary material for: Intraparenchymal Enzyme Injections in Islet Isolations With Incomplete Ductal Perfusion of Enzymes
Source: Transpl Int. 2025 Apr 30;38:13507. doi: 10.3389/ti.2025.13507 (PMC12074925; doi:10.3389/ti.2025.13507)
Supplement: Supplementary file 2 [file DataSheet1.PDF]

Supplementary Methods

Pancreas procurement and characteristics

Pancreases obtained from organ donors were allocated to patients on the islet transplantation waiting list by Eurotransplant. Donor pancreases were preserved in Belzer UW cold storage solution and stored on ice during transport to the human isolation laboratory at the Leiden University Medical Center (allogeneic islet isolation).

For autologous islet transplantation, a total pancreatectomy was performed after work-up by a multidisciplinary team, having considered patient histories, including episodes of pancreatitis and previous pancreatic surgery. During pancreatectomy, the blood supply of the pancreas was severed just prior to back-table flushing of the organ with cold Ringers' Acetate supplemented with 7mM of calcium chloride (CaCl2). The pancreas was then stored in this Ringers' Acetate solution on ice and transported to our human isolation laboratory (autologous islet isolation).

Relevant patient, donor , procurement and islet isolation characteristics such as age, body mass index (BMI), sex, donation after circulatory death (DCD), donation after brain death (DBD), warm ischemia time (WIT), cold ischemia time (CIT) and islet isolation techniques were recorded for analysis.

Islet isolation

After dissection of non-pancreatic tissue, the main (or accessory) pancreatic duct was identified and ligated at the duodenal orifice. With the standard retrograde cannulation (RC) technique (Figure 1: A1), the pancreatic duct was cannulated followed by the infusion enzyme solution containing Collagenase NB1, Neutral Protease and methylene blue. If RC proved challenging, the pancreas was dissected at the neck and combined ante- and retrograde cannulation was performed for enzyme perfusion (Figure 1: A2). After full distention, the pancreas was cut and the pieces were transferred to a digestion chamber with silicon nitride marbles for further digestion, separation from exocrine tissue and collection.

Intraparenchymal injection

For intraparenchymal injection a 25-30 gauge BD Microlance Needle was attached to either a 5ml BD Plastipak Syringe or a silicone infusion line driven by a peristaltic pump to support either manual or automatic continuous infusion of enzyme solution. IPI was typically started manually as the peristaltic pump would be used for intraductal infusion. However, if intraductal enzyme infusion was not possible, the peristaltic pump could also be used for IPI. The needle was inserted into the hypoperfused areas of the pancreas, and care was taken to change the injection site regularly in order to distribute the enzyme solution evenly. The tissue areas distended upon injection of the enzyme solution.

Data handling and analysis

Data were excluded from this study if the information regarding digestion was incomplete, the islet isolation was interrupted due to a technical error, the islet isolation deviated from the standard protocol (e.g. during a PRISM isolation[1]) or the isolation was performed on an explanted allogeneic pancreas (allo-auto transplantation).[2] All isolations in which IPI was performed (including partial IPI), were labelled as such. Donor age and pancreas weight before isolation were considered confounding variables based on literature and expert knowledge, and were adjusted for in linear regression models. Data distribution was assessed for normality in histograms and reported as mean ± standard deviation or median with interquartile range. Non-normally distributed data were analyzed using Mann-Whitney U tests. Data were analyzed in Rstudio with alpha set at 0.05.

Reagents and materials
Ringers' Acetate: B. Braun, Melsungen, Germany
Belzer UW cold storage solution: Bridge to Life, Wandsworth, England
Collagenase NB1: Serva Electrophoresis GmbH, Heidelberg, Germany; Nordmark Biochemicals, Uetersen, Germany
Neutral Protease: Serve Electrophoresis GmbH; Nordmark Biochemicals
Methylene blue: Provepharm, Marseille, France
Silicon nitride marbles: Biorep Diabetes Inc, Miami, United States
25-30 gauge BD Microlance Needle: BD, Vianen, the Netherlands
5ml BD Plastipak Syringe: BD
Peristaltic pump: Masterflex, Metrohm, Barendrecht, The Netherlands

Video S1. Video of a demonstration of intraparenchymal injections (IPI) in pancreatic islet isolation. In this pancreas, there was no indication for IPI on the basis of hypoperfusion. This pancreas was rejected for use in clinical islet transplantation and there was consent for use in research. Video created by D.J.C. Voice-over by D.J.C. Video duration: 1:00 minutes. File size: 24.8MB.

Supplementary Results

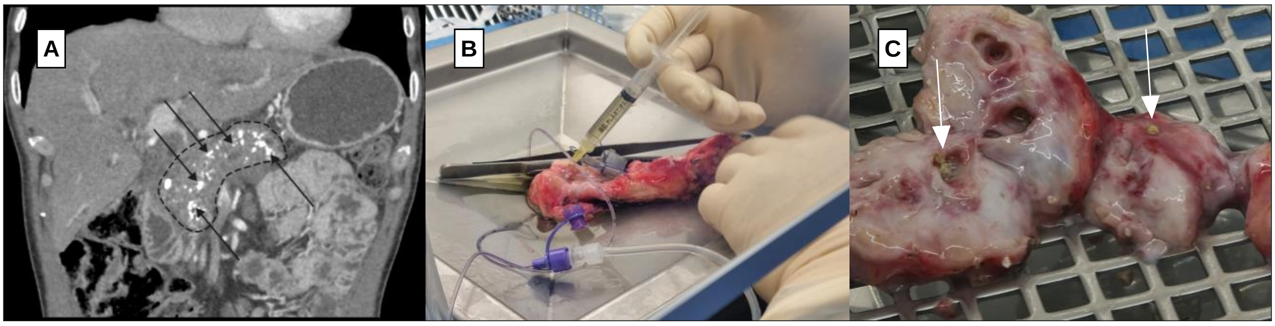

Figure S1. A) Computed tomography image of the abdomen in a patient with 1.5 years of chronic pancreatitis. The pancreas (indicated with black dotted line) is enlarged and calcifications (white spots, black arrows) are diffusely present. B) Pancreas after pancreaticojejunostomy of a patient with 2 years of chronic pancreatitis. Digestive enzymes are injected in the pancreas head. C) Fibrotic pancreas of a patient with 6 years of chronic pancreatitis. The pancreatic duct is enlarged. Calcifications are indicated with white arrows. Images are shown with consent of the patients.

Table S1. Donor characteristics of pancreases for allogeneic islet transplantation	
**Number of pancreases**	253
**Age (years)**	47.9 ± 12.7
**Female sex**	115 (45.5)
**Body mass index (kg/m²)**	27.3 ± 5.11
**Weight pancreas before isolation (gram)**	105 ± 25.8
**Donation type**	
**DBD**	145 (57.3)
**DCD-3**	78 (30.8)
**DCD-5**	30 (11.9)
**Warm ischemia time (minutes)**	0 (0 – 15.0)
**Cold ischemia time (minutes)**	467 ± 191
**Isolation technique**	
**RC**	206 (81.4)
**ARC**	18 (7.1)
**RC with IPI**	12 (4.7)
**ARC with IPI**	17 (6.7)
**Transplanted**	66 (26.1)

Data shown as mean ± standard deviation, median (interquartile range) or number (percentage). DBD: donation after brain death, DCD: donation after circulatory death, RC: retrograde cannulation, ARC: ante- and retrograde cannulation, IPI: intraparenchymal injection.

Table S2. Patient characteristics of pancreases for autologous islet transplantation	
	**Total**
**Number of pancreases**	26
**Age (years)**	45.5 ± 14.9
**Female sex**	17 (65.4)
**Body mass index (kg/m²)**	24.0 ± 4.07
**Weight pancreas before isolation (grams)**	76.4 ± 32.1
**Chronic pancreatitis**	22 (84.6)
**Duration of chronic pancreatitis (years)**	3.50 (2.00 – 7.75)
**Previous pancreatic surgery**	
**Frey**	6 (23.1)
**Whipple/PPPD**	2 (7.7)
**Lateral PJ**	3 (11.5)
**Distal pancreatectomy**	1 (3.8)
**Warm ischemia time (minutes)**	7.00 (2.82 – 9.50)
**Cold ischemia time (minutes)**	105 (92.3 – 126)
**Isolation technique**	
**RC**	3 (11.5)
**ARC**	1 (3.8)
**RC with IPI**	10 (38.5)
**ARC with IPI**	6 (23.1)
**IPI only**	6 (23.1)
**Transplanted**	23 (88.5)

Data shown as mean ± standard deviation, median (interquartile range) or number (percentage). PPPD: pylorus preserving pancreatoduodenectomy, PJ: pancreatico-jejunostomy, RC: retrograde cannulation, ARC: ante- and retrograde cannulation, IPI: intraparenchymal injection.

Table S3. Isolation outcomes.					
	**Total**	**With IPI**	**Without IPI**	**Mean difference (95% CI)**	**P**
**Allogeneic pancreas – all isolations**					
**Number of pancreases**	253	29	224		
**Digestion (%pt)**	86.1 ± 10.0	88.8 ± 7.21	85.8 ± 10.3	3.02 (-0.71 – 6.75)†	0.11
**Islet yield per gram pancreas (IEQ/g)**	4,730 ± 2,830	4,730 ± 3,260	4,730 ± 2,780	7 (-1,073 – 1,088)†	0.99
**Islet yield *($x10^3$ IEQ)***	483 ± 294	472 (264 – 625)	415 (252 – 670)	-	0.78
**Allogeneic pancreas – RC isolations**					
**Number of pancreases**	218	12	206		
**Digestion (%pt)**	86.5 ± 10.2	88.4 ± 9.16	86.4 ± 10.3	2.38 (-3.43 – 8.18)	0.42
**Islet yield per gram pancreas (IEQ/g)**	4,740 ± 2,740	3,980 ± 2,210	4,780 ± 2,760	-801 (-2,395 – 793)	0.33
**Islet yield *($x10^3$ IEQ)***	481 ± 277	486 (226 – 597)	427 (256 – 676)	-	0.74‡
**Allogeneic pancreas – ARC isolations**					
**Number of pancreases**	35	17	18		
**Digestion (%pt)**	83.8 ± 8.48	89.1 ± 5.76	78.9 ± 7.75	10.04 (5.99 - 14.08)	<0.001
**Islet yield per gram pancreas (IEQ/g)**	4,220 (2,010-5,970)	4,620 (3,380 – 6,950)	3,680 (2,000 – 5,690)	-	0.44‡
**Islet yield *($x10^3$ IEQ)***	401 (248-618)	456 (280 – 625)	361 (215 – 464)	-	0.16‡
**Autologous pancreas – all isolations**					
**Number of pancreases**	26	22	4		
**Digestion (%pt)**	77.4 ± 19.7	81.4 ± 15.5	55.0 ± 27.4	26.4 (7.82 – 45.02)	0.01
**Islet yield per gram pancreas (IEQ/g)**	4640 (2,920 – 7,190)	5,540 (3,100 – 7,330)	2,570 (1,870 – 3,230)	-	0.05‡
**Islet yield *($x10^3$ IEQ)***	295 (211 – 446)	333 (214 – 517)	187 (76.6 – 289)	-	0.13‡

† Corrected for age of the donor. ‡ Mann-Whitney U test. Data shown as mean ± standard deviation, median (interquartile range) unless otherwise indicated. IEQ: islet equivalent, RC: retrograde cannulation, ARC: ante- and retrograde cannulation, IPI: intraparenchymal injection, %pt: percentage point, CI: confidence interval.

Table S4. Islet isolation procedures with only intraparenchymal injections										
**Sex**	**Age (years)**	**Indication TP**	**Duration CP (years)**	**Prior surgery**	**Pancreas weight (grams)**	**Digestion (%)**	**Purity (%)**	**Islet yield (IEQ)**	**Islet yield by bodyweight (IEQ/kg)**	**Transplanted**
F	37	CP	34	Lateral PJ	33.8	90.2	10	247,826	4130	Yes
F	28	CP	5	Whipple/PPPD	11.2	97.3	55	239,103	3312	Yes
M	32	CP	6	Frey	69.0	77.4	5	217,391	3260	No, high endotoxin
F	48	CP	2	Frey	82.6	79.9	9	536,902	9503	Yes
M	29	CP	13	Lateral PJ	96.5	78.2	48	704,891	7267	Yes
F	21	CP	12	Frey	72.4	83.3	9	190,217	2972	Yes

TP: total pancreatectomy, CP: chronic pancreatitis, PJ: pancreaticojejunostomy, PPPD: pylorus-preserving pancreatoduodenectomy, IEQ: islet equivalent, F: female, M: male.

References

1. Doppenberg JB, Engelse MA, de Koning EJP. PRISM: A Novel Human Islet Isolation Technique. Transplantation. 2022;106(6):1271-8.
2. Nijhoff MF, Dubbeld J, van Erkel AR, van der Boog PJM, Rabelink TJ, Engelse MA, et al. Islet alloautotransplantation: Allogeneic pancreas transplantation followed by transplant pancreatectomy and islet transplantation. Am J Transplant. 2018;18(4):1016-9.
